# Biochemical and Genetical Responses of* Phoenix dactylifera* L. to Cadmium Stress

**DOI:** 10.1155/2017/9504057

**Published:** 2017-10-19

**Authors:** Fahad Al-Qurainy, Salim Khan, Mohamed Tarroum, Mohammad Nadeem, Saleh Alansi, Aref Alshameri

**Affiliations:** Department of Botany and Microbiology, College of Science, King Saud University, Riyadh 11451, Saudi Arabia

## Abstract

The cadmium (Cd), a heavy metal, causes toxicity, which leads to hampering the growth and development of the plant. The molecular and biochemical approaches were used for the investigation of antioxidant system response and genotoxicity in date palm (*Phoenix dactylifera *L.) cv. Sagai in pot experiment having Cd. The root length was more affected than the shoot length as more accumulation of Cd occurs in roots. Fresh weights of root and shoot were reduced significantly in treated plants as compared to the control. The proline content was increased at low concentration of Cd (300 *µ*M-CdCl_2_) than the medium and high concentrations (600 and 900 *µ*M-CdCl_2_), respectively. The thiobarbituric acid reactive substances (TBARS) content was increased at 600 and 900 *µ*M-CdCl_2_ compared to the plants treated at 300 *µ*M-CdCl_2_ and controls. Antioxidant enzymatic assay was performed under Cd stress and compared with control plants. The catalase (CAT) and superoxide dismutase (SOD) activities were found to be high in plants treated with CdCl_2_ at 300 *µ*M compared to at 600 and 900 *µ*M-CdCl_2_, respectively. The genotoxicity of Cd was assessed using the inter-simple sequence repeat (ISSR) marker where all treated and control plants were clustered into three main groups based on genetic similarity.* P. dactylifera *plants were found to be more divergent at high Cd stress as compared to control and plants treated at low concentration of Cd.

## 1. Introduction 

Heavy metals are inorganic chemical constituents which have mutagenic, cytotoxic, and genotoxic effects on plants, humans, and animals [1–4]. Cadmium (Cd) is considered in the top ten list of hazardous compounds by the Agency for Toxic Substances and Disease Registry (https://www.atsdr.cdc.gov). Among the heavy metals, Cd is toxic to animals and plants due to its nonessentiality in living organism [5]. The food, which has contamination of Cd, is the main source of entry to humans via the food chain [6]. The high uptake of the bivalent cations to the aerial parts of the plant shifts its cellular phosphorylation state and causes a range of physiological disturbances and oxidative stresses in the cell [7, 8]. The plant may get the Cd from the phosphate fertilizer or sewage sludge as these are used for soil enhancement [9–11]. The older plants accumulate more heavy metals at their inactive sites such as cell walls and vacuoles [12]. The Cd inhibits the growth of lateral root formation in plants while the main root became brown, rigid, and twisted [13, 14]. The restricted activity of photosynthesis and chlorosis has been seen in many plant species under Cd stress [15, 16]. It interferes with the uptake and transport of various elements from the soil [17]. The functions and stability of the cell were affected under Cd stress as it binds to enzymes and membranes [18]. 

The mutagenic and cytotoxic nature of Cd causes DNA damage by producing reactive oxygen species [12]. Moreover, Cd binds to DNA bases and inhibits DNA mismatch repair [19, 20]. The genotoxic effect was varied among the plant organs of lettuce and tobacco as more effects were seen in the root and no changes were observed in the leaf [21, 22]. The genotoxicity of Cd varies from organ to organ as more DNA damage was seen in the roots of* Nicotiana tabacum* but no effect was seen in the leaves [22]. The oxidative DNA damage, chromosomal aberrations, DNA strand breaks, and induction of micronuclei have been observed* in vivo* and* vitro* under Cd stress [23]. Different molecular markers such as random amplified polymorphic DNA (RAPD) [24], microsatellite (simple sequence repeat, SSR) [21], and inter-simple sequence repeat (ISSR) [25] have been used to assess the genotoxicity in plants caused by various heavy metals.

The cadmium is dispersed in environment through mining, smelting, phosphate fertilizers, sewage sludge, Ni-Cd batteries, plating, pigments, and plastics items. The environmental Cd goes in the soil with rain water and is taken up by the plant which then enters the food chain. The different level of Cd has been reported in the fruits of* P. dactylifera* from different countries including Saudi Arabia, Egypt, Oman, and Pakistan [26–29]. However,* P. dactylifera* survives under extreme abiotic stresses, including high temperature, relatively high soil salinity levels, and drought [30–33]. The response of antioxidant enzymes in general to metals and Cd can also vary in various tissues and among plant species [6, 34, 35]. The cadmium toxicity also causes oxidative damage in plants through generation of reactive oxygen species ROS [36, 37]. However, antioxidant system plays an important role in removal of ROS and provides tolerance to plants under abiotic stresses. The stress marker “proline” maintains the cellular homeostasis in the plants under Cd stress. The present study focuses on Cd tolerance in* P. dactylifera* using the biochemical and genetic approaches.

## 2. Materials and Methods

The pot experiment was performed in a growth chamber for Cd stress treatment on Sagai cultivar of* P. dactylifera*. The same size of pots was filled in a ratio (3 : 1) with a mixture of sand and peat moss. The seeds were washed with tap water three times and thereafter surface-sterilized with sodium hypochlorite solution (4.0% available chlorine) for 10 min. Further, these were rinsed thoroughly three times with autoclaved distilled water. One seed per pot was sown in plastic pot, watered at regular interval to keep moisture. The exposure of Cd was given to 2-month-old plants in solution form (100 ml per pot after two-week time intervals) to see its effect on antioxidant system and growth development. The three concentrations of CdCl_2_ were used as low (T1-300 *µ*M), medium (T2-600 *µ*M), and high (T3-900 *µ*M) to treat the 2-month-old plants. The relative humidity (72%) and photoperiod with temperature 26-27°C for 16 h per day were maintained in the growth chamber for proper growth of the plants. The Cd treated and control plants were harvested after 90 days of treatment to perform molecular and biochemical parameters.

### 2.1. Estimation of Biomass

Fresh roots and leaves weights were measured after 90 days of Cd treatment. The shoot and root length were also measured.

### 2.2. Genomic DNA Isolation and Evaluation of Genotoxicity

The genomic DNA was isolated using the protocol developed by Khan et al. [38]. The PCR reaction was performed in 25 *µ*L volume. The PCR bead (master mixture, GE health care) was used for the amplification of genomic DNA extracted from the control as well as treated samples. ISSR marker was used to assess the genotoxicity caused by Cd stress. The doubled distilled water was added in the master mixture followed by ISSR primer (Table 1) and template DNA. The PCR program was set in Veriti 96-well Thermal Cycler (Applied Biosystems, Singapore). First denaturation at 94°C for 3 min, followed by 38 cycles at 94°C for 1 min, 48°C for 1 min, 72°C for 1 min, and final extension at 72°C for 5 min, was carried out for the amplification. Agarose gel (1.3%) was prepared in 1x TBE buffer solution for electrophoresis.

### 2.3. Proline Estimation

The proline estimation was performed according to the protocol of Hanson et al. [39]. The fresh leaf samples of 0.3 g were ground in 10 ml of sulphosalicylic acid (30% aqueous). The mixture was centrifuged at 9000 ×g for 15 min and, thereafter, 2 ml of supernatant was taken in another glass tube. An equal volume of acid ninhydrin and acetic acid were added in the above step and incubated for 1 h in boiling water. The reaction was stopped by putting it into the ice bath. The mixture was taken out from the ice bath and 4 ml of toluene was added and vortexed for 20 s. The upper phase was taken for the estimation of the proline using the spectrophotometer at 520 nm (Model UB-1800, Shimadzu, Japan).

### 2.4. Total Chlorophyll

Total chlorophyll was estimated in the fresh leaves according to the method of Arnon [40]. Leaves were chopped in small pieces (0.1 g) and 10 ml of dimethyl sulfoxide (DMSO) was added to each test tube. The incubation was completed at 65°C in oven for 120 minutes to release the whole chlorophyll in DMSO. The tubes were taken out from the oven and absorbance of the solution was recorded at 663 and 645 nm on a UV-vis spectrophotometer (Model UB-1800, Shimadzu, Japan). The content of chlorophyll was calculated as mg/g fresh weight.

### 2.5. Superoxide Dismutase (SOD) (EC 1.15.1.1)

Dhindsa et al. [41] method was used for the activity assay of superoxide dismutase. The fresh leaf samples (0.05 g) were chopped in small pieces and ground in 2.0 ml of extraction buffer containing 0.5 M phosphate buffer (pH 7.3), 0.3 mM-EDTA, 1% PVP (w/v), and 1% Triton x100 (w/v). The supernatant was taken after centrifugation for the assay of SOD activity. The absorbance of the reaction mixture along with blank was read at 560 nm, using the UV-vis spectrophotometer. A 50% reduction in color was considered as one enzyme unit (EU), and the activity was expressed in EU mg^−1^ protein min^−1^.

### 2.6. Catalase (CAT) (EC 1.11.1.6)

The CAT activity was determined by estimating H_2_O_2_ degradation according to the method of Aebi [42]. The reaction was performed in 3.0 ml of reaction mixture containing 10 mMH_2_O_2_, 100 mM potassium phosphate buffer solution (pH 7.0), and 100 *µ*l of enzyme extract. The decrease in absorbance of H_2_O_2_ was recorded at 240 nm using the UV-vis spectrophotometer (Model UB-1800, Shimadzu, Japan). The enzyme activity was calculated using the extinction coefficient (0.036 mM^−1^ cm^−1^). One unit of CAT determines the amount necessary to decompose 1 *µ*mol of H_2_O_2_ per min at 25°C.

### 2.7. Thiobarbituric Acid Reactive Substances (TBARS)

The TBARS content was estimated in fresh leaves using the method described by Cakmak and Horst [43]. 0.5 g of fresh leaves was ground in 5.0 ml of 0.1% (w/v) trichloroacetic acid (TCA) at 4°C. The reaction mixture was taken in falcon tube and centrifuged at 12,000 ×g for 5 min. The 4.0 ml of 0.5% (w/v) TBA in 20% (w/v) TCA was added in 1.0 ml of supernatant taken from the above step. The mixture was kept for 30 min at 90°C in water bath. After incubation of mixture, the reaction was terminated by keeping it on ice bath. The reaction mixture was centrifuged at 10,000 ×g for 5 min, and absorbance of the supernatant was taken at 532 and 600 nm on a spectrophotometer (Model UB-1800, Shimadzu, Japan).

The amount of TBARS was calculated using an extinction coefficient of 155 mM cm^−1^ as follows:

(1) $$\begin{array}{c} \text{TBARS}\left(\;\text{nmol }{\text{g}}^{-1}\text{ fw}\;\right)=\frac{\left({\text{A}}_{532}-{\text{A}}_{600}\right)\times \text{V}\times 1000}{155\left(\text{extinction coff.}\right)\times W}, \end{array}$$

where

 
*A*_532_ is absorbance at 532 nm
 
*A*_600_ is absorbance at 600 nm
 
*V* is extraction volume
 
*W* is fresh weight of tissues and methods.

### 2.8. Determination of Elements in Leaves and Roots

The leaf and root powders were digested according to the method developed by Price [44]. The fine powder of leaves and roots (200 mg) was taken in TECAM digestion flask in which 0.5 ml of sulphuric acid, 1.0 ml of perchloric acid, and 5.0 ml of nitric acid were added. The flasks were heated at 110°C and further temperature was increased gradually to 330°C. The samples were taken out and cooled down. Thereafter, samples were transferred to 50 ml calibrated flask and volume was made up with distilled water. The content of Cd along with other minerals (Mg, Ca, and K) was measured by inductively coupled plasma atomic emission spectroscopy (ICP-AES).

### 2.9. Data Analysis

One-way analysis of variance (ANOVA) was used for data analysis obtained from treated and untreated samples. The significant differences among the treatment means were evaluated using Duncan's multiple range test [45]. ISSR marker was used for the evaluation of genotoxicity caused by Cd stress. All primers data were combined into a binary matrix as absence (0) or presence (1) of the bands on agarose gel. The similarity matrix value was calculated using the NTSYS.pc software version 2.21 package between the control and treated* P. dactylifera* plants [46].

## 3. Results and Discussion

**Figure 1:**
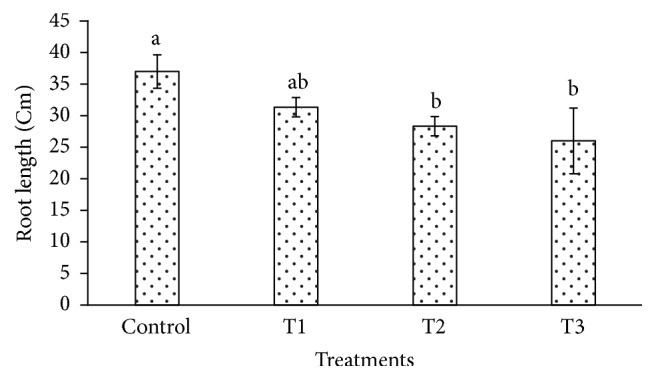
Root length variation in* Phoenix dactylifera* in response to Cd stress. Data are means of three replicates ± standard deviation; symbols indicated by different letters on bars represent the significant values according to Duncan's test (*p* < 0.05).

**Figure 2:**
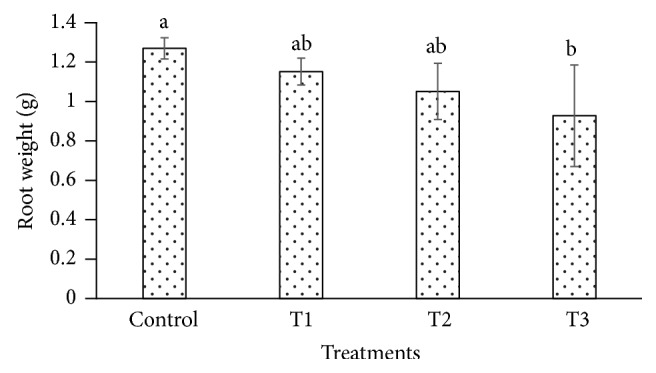
Root weight variation in* Phoenix dactylifera *in response to Cd stress. Data are means of three replicates ± standard deviation; symbols indicated by different letters on bars represent the significant values according to Duncan's test (*p* < 0.05).

**Figure 3:**
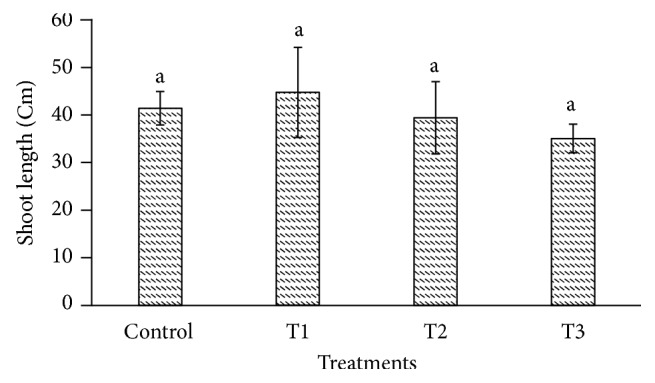
Changes in shoot length in response to Cd stress in* Phoenix dactylifera*. Data are means of three replicates ± standard deviation; symbols indicated by different letters on bars represent the significant values according to Duncan's test (*p* < 0.05).

**Figure 4:**
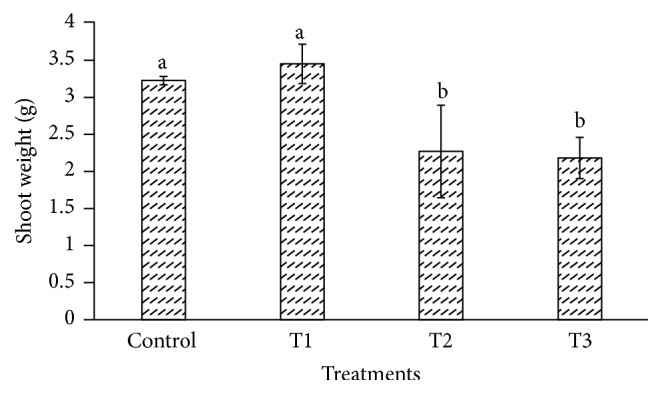
Shoot weight in* Phoenix dactylifera* plant in response to Cd stress. Data are means of three replicates ± standard deviation; symbols indicated by different letters on bars represent the significant values according to Duncan's test (*p* < 0.05).

The heavy metals cause toxicity and also generate oxidative stress in plant cell by interfering with the antioxidant defense system [47–49]. The cadmium affects the biomass and plant height in plant species such as* Gossypium hirsutum* and* Cichorium pumilum* [50, 51]. In our study,* P. dactylifera *plants were harvested at 90 days after Cd treatment to investigate its effect using the biochemical and molecular approaches. The root length and weight of* P. dactylifera* were decreased under Cd stress in a dose dependent manner (Figures 1 and 2). The root length was decreased significantly as 31.333, 28.333, and 26 cm at 300, 600, and 900 *µ*M-CdCl_2_ in relation to control (37 cm). Similarly, root weight was also decreased significantly as 1.146, 1.045, and 0.922 g at 300, 600, and 900 *µ*M-CdCl_2_ treatments as compared to control (1.263 g), respectively. However, shoot length was less affected at all Cd concentrations applied in the experiment. Shoot weight was decreased as 2.256 g and 2.168 g at 600 and 900 *µ*M-CdCl_2_ as compared to control (3.206 g) but result was found nonsignificant at low concentration of Cd application (Figures 3 and 4).

**Figure 5:**
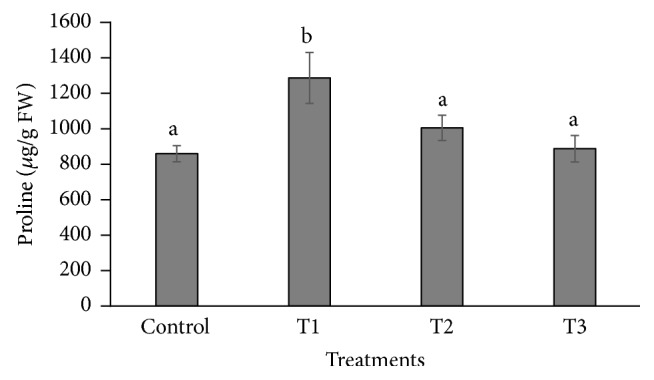
Changes in proline contents in response to Cd stress in* Phoenix dactylifera* leaves. Data are means of three replicates ± standard deviation; symbols indicated by different letters on bars represent the significant values according to Duncan's test (*p* < 0.05).

**Figure 6:**
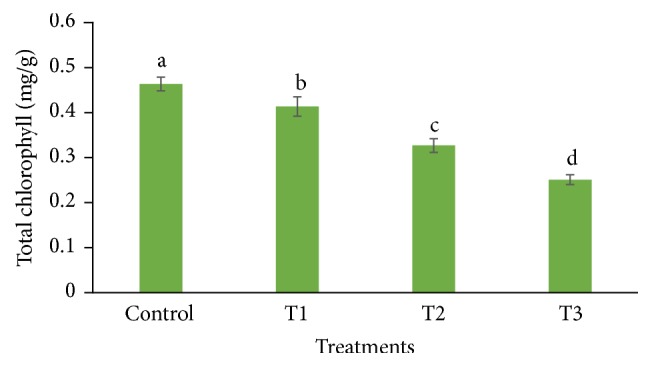
Total chlorophyll in response to Cd stress in* Phoenix dactylifera* leaves. Data are means of three replicates ± standard deviation; symbols indicated by different letters on bars represent the significant values according to Duncan's test (*p* < 0.05).

The proline content was increased (1283.055 *µ*g/g FW) significantly in leaves of* P. dactylifera* plants treated at 300 *µ*M-CdCl_2_ as compared to control (856.746 *µ*g/g FW) (Figure 5). However, the response was found nonsignificant at 600 and 900 *µ*M-CdCl_2_ as compared to controls. The proline content was increased in* Brassica juncea*,* Groenlandia densa*, and* Medicago sativa* as the Cd concentration increased in the treatment [52–54]. Total chlorophyll content was decreased in the leaf of* P. dactylifera* plant under Cd stress in a dose dependent manner in relation to controls (Figure 6). The chlorophyll content decreased in various plant species such as strawberry, faba bean, pakchoi, and mustard as the Cd concentration increased in the treatment [55–57].

**Figure 7:**
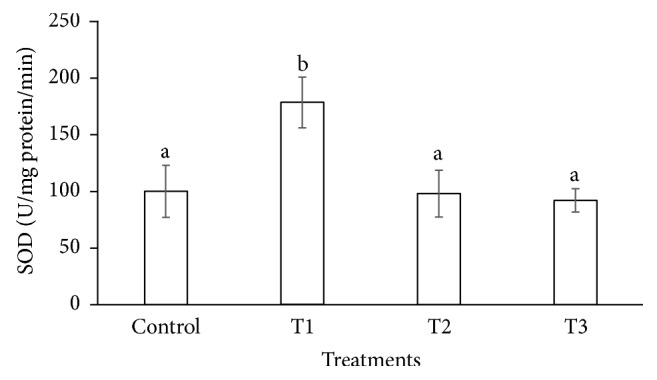
Superoxide dismutase activities in response to Cd stress in* Phoenix dactylifera* leaves. Data are means of three replicates ± standard deviation; symbols indicated by different letters on bars represent the significant values according to Duncan's test (*p* < 0.05).

**Figure 8:**
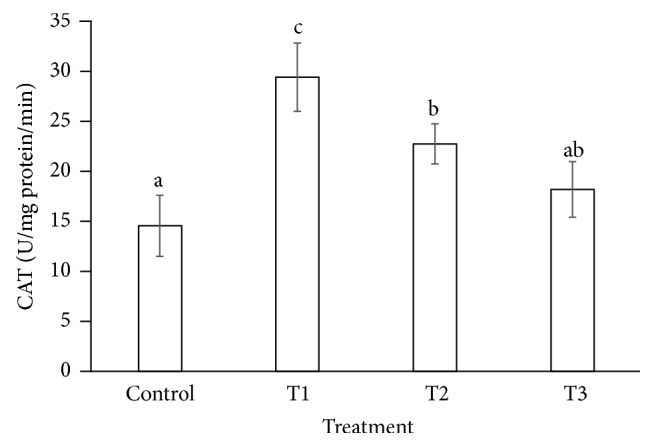
Catalase activities in* Phoenix dactylifera* leaves in response to Cd stress. Data are means of three replicates ± standard deviation; symbols indicated by different letters on bars represent the significant values according to Duncan's test (*p* < 0.05).

The antioxidant enzymes play important role under abiotic and biotic stresses to protect the cells from oxidative damage. The SOD and CAT activities were increased (178 and 29.400 U/mg protein/min) significantly in the leaf of* P. dactylifera* plants treated at 300 *µ*M-CdCl_2_ (Figures 7 and 8) in relation to controls (99.770 and 14.541 U/mg protein/min). However, SOD activity was found nonsignificant at 600 and 900 *µ*M-CdCl_2_ in relation to control. Similarly, CAT and SOD activities were enhanced at low concentration of Cd stress, whereas it was decreased at high concentration of Cd in* Lemna polyrhiza* [58]. The CAT activity was decreased in many plant species under Cd stress including* Amaranthus lividus *[59],* Phaseolus aureus* [60],* Lemna minor* [61], and hybrid poplar [62] under the Cd stress. The SOD activity was increased under Cd stress in bean [63], pea [64], and wheat [65], whereas, in other plant species including* Pisum sativum *[65],* Phaseolus vulgaris *[66], and* Helianthus annuus *[67], the activities of both CAT and SOD were decreased.

**Figure 9:**
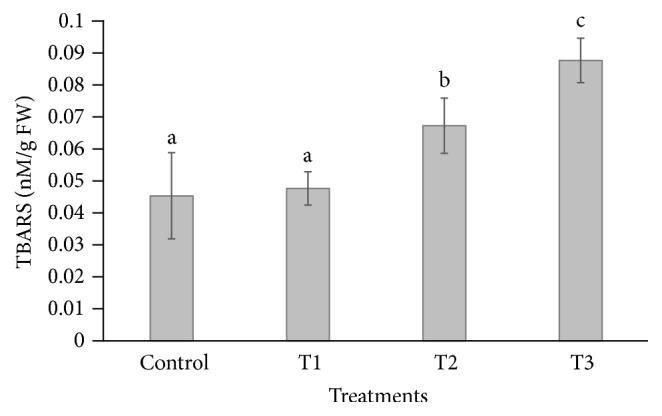
Changes in TBARS contents in* Phoenix dactylifera* leaves in response to Cd stress. Data are means of three replicates ± standard deviation; symbols indicated by different letters on bars represent the significant values according to Duncan's test (*p* < 0.05).

The TBARS content was increased (0.067 and 0.876 nM/g FW) significantly in* P. dactylifera* leaves at 600 and 900 *µ*M-CdCl_2_ concentrations as compared to control (0.045 nM/g FW); however, the result was found nonsignificant at 300 *µ*M-CdCl_2_ as compared to the controls (Figure 9). Similarly, TBARS content was also increased under Cd stress in leaf and root of strawberry as reported by Muradoglu et al. [55]. Cd caused an enhancement of lipid peroxidation in* Pisum sativum *[68],* H. annuus *[67], and* Phaseolus vulgaris *[66].

The content of Cd along with other minerals was estimated in leaves and roots of* P. dactylifera *plants under various concentration of Cd treatment along with control plants. In our study, the accumulation of heavy metals was more in root organ as compared to the leaf organ (Table 2). As the Cd concentration increased in the treatment, the accumulation of Cd increased both in leaf and in root organs, and more accumulation (0.187 and 0.223 mg/g DW) was observed at high Cd concentrations (900 *µ*M-CdCl_2_) as compared to controls (0.063 and 0.092 mg/g/DW) (Table 2). Our result was lined with previous work as carried out by Gichner et al. [68] as the roots have accumulated more Cd content than the above ground parts. The more Cd content was also accumulated in roots of* Olea europaea* L. cv. Chemlali and* Helianthus annuus* L. cv. Oleko under Cd stress (Zouari et al., 2016; De Maria et al., 2013) [55, 69, 70]. However, Pillay et al. [28] reported that the accumulation of Cd in leaves of* P. dactylifera* was more than the root organ. Similarly, the accumulation of magnesium (Mg), calcium (Ca), and potassium (K) was also decreased in leaves and roots of* P. dactylifera* in a dose dependent manner. The content of manganese (Mn), potassium (K), and zinc (Zn) was decreased in roots as well in shoots of wheat at high level of Cd toxicity [71]. The mineral contents of iron (Fe), Mg, K, and Ca were decreased in wheat shoots in a dose dependent manner under excess Cd stress [72]. However, Cd ions may compete with Ca, Mg, or iron (Fe) in their transport across membranes [73] which may cause mineral deficiency in these organs.

The individual* P. dactylifera* leaf was taken for the evaluation of genotoxicity under Cd stress. Fifteen ISSR primers were used for the evaluation of genotoxicity. Out of fifteen primers, twelve primers produced bright and reproducible bands which were used further for the comparison between and among the control and treated plants. In other ISSR primers, the obtained ISSR profiles were different at many loci between treated and control plants. The more polymorphism was observed among the treated plants at medium and higher concentration of Cd and it might be due to the mutations produced by Cd toxicity. Similarly, in other plant species, the polymorphism was also detected under Cd stress using the molecular markers [25, 74–76]. However, some primers gave faint and nonreproducible bands in PCR amplification and were excluded from the data analysis. Genetic similarity was calculated among the control and treated plants using the NTSYS.pc software version 2.21. In our study, the plants treated at 300 *µ*M-CdCl_2_ showed more genetic similarity (98.81%) to control plants. The genetic similarity (96.43%) was found among the plants treated at 300, 600, and 900 *µ*M-CdCl_2_ and controls. A very low genetic similarity (92.86%) was observed between the plants treated at 300 and 900 *µ*M-CdCl_2_.

**Figure 10:**
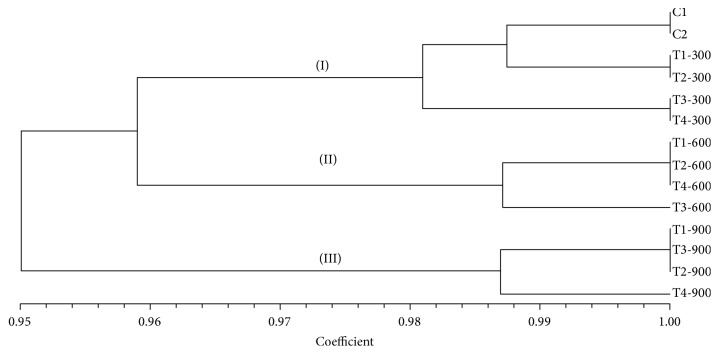
Dendrogram showing the clustering of* P. dactylifera* plants based on genetic similarity controls (C1, C2); T-300 (300 *µ*M-CdCl_2_); T-600 (600 *µ*M-CdCl_2_); T-900 (900 *µ*M-CdCl_2_).

All plants were clustered into three main groups according to genetic similarity (Figure 10). In the first group, controls and plants treated at low Cd concentration were clustered. In second and third groups, medium and high concentrations treated plants were clustered, respectively. Thus, genetic variation was produced in* P. dactylifera* plants at different concentration of Cd treatment and more effect was observed at its high concentration. The highest percentage of polymorphism was detected in the roots and shoots of* Trifolium repens* under As and Cd treatment as they caused genotoxicity and it was related to their concentrations [77]. The genotoxicity was assessed in germinated seeds of* Eruca sativa* under the Zn, Pb, and Cd stresses using the ISSR marker [25], whereas Cd showed more genotoxicity than the other two heavy metals. Thus, in conclusion,* P. dactylifera* plant grown under high concentration of Cd showed less genetic similarity, reduction in biomass, and chlorophyll content and lower root length than the plant under low Cd stress and controls. However, the enzymatic activities of CAT and SOD were found to be high in plants grown at low Cd stress than the high Cd stress and controls.

## Figures and Tables

**Table 1:** List of ISSR primers used to assess genotoxicity among control and treated plants.

Primer code	Primer sequence (5′-3′)
OP-1	AGTCAGTCAGTCAGTC
OP-2	AGAGAGAGAGAGAGAGCTC
OP-3	GAG AGA GAG AGA GAG AA
OP-4	CTC TCT CTC TCT CTC TT
OP-5	CTC TCT CTC TCT CTC TA
OP-6	CTC TCT CTC TCT CTC TG
OP-7	CAC ACA CAC ACA CAC AT
OP-8	CAC ACA CAC ACA CAC AA
OP-9	CAC ACA CAC ACA CAC AG
OP-10	GTG TGT GTG TGT GTG TA
OP-11	GTG TGT GTG TGT GTG TC
OP-12	GTG TGT GTG TGT GTG TT
OP-13	TCT CTC TCT CTC TCT CA
OP-14	TCT CTC TCT CTC TCT CC
OP-15	TCT CTC TCT CTC TCT CG

**Table 2:** Mineral content (mg/g dry weight) in leaves and roots of treated and control plants of *Phoenix dactylifera* under Cd stress.

Treatments (CdCl_2_)	Leaf				Root			
	Mg	Ca	K	Cd	Mg	Ca	K	Cd
Control (0 *µ*M)	7.063 ± 0.097^c^	40.29 ± 1.44^b^	64.83 ± 0.99^d^	0.063 ± 0.0041^a^	2.383 ± 0.037^d^	31.29 ± 1.32^c^	38.38 ± 1.32^c^	0.092 ± 0.0015^a^
300 *µ*M	6.466 ± 0.075^b^	30.77 ± 1.24^a^	60.4 ± 0.96^c^	0.121 ± 0.0076^b^	1.593 ± 0.025^c^	25.4 ± 0.93^b^	35.75 ± 1.08^b^	0.118 ± 0.001^b^
600 *µ*M	5.64 ± 0.13^a^	29.41 ± 1.26^a^	57.29 ± 1.29^b^	0.139 ± 0.0041^c^	1.473 ± 0.035^b^	24.4 ± 0.65^b^	27.21 ± 0.45^a^	0.172 ± 0.0041^c^
900 *µ*M	5.53 ± 0.95^a^	28.32 ± 1.19^a^	53.93 ± 1.66^a^	0.187 ± 0.0046^d^	1.386 ± 0.0305^a^	19.6 ± 0.85^a^	26.21 ± 0.40^a^	0.223 ± 0.003^d^

Mean of three replicates ± SD. The letters a, b, c, and d represent the significant values according to Duncan's test (*p* < 0.05).
